# Endometrial Autologous Intrauterine Platelet-Rich Plasma (PRP) Instillation Treatment and Its Potential on In Vitro Fertilization (IVF): Narrative Review

**DOI:** 10.7759/cureus.101771

**Published:** 2026-01-18

**Authors:** Vaia Sarli, Emmanouil Kalampokas, Theodoros Kalampokas

**Affiliations:** 1 3rd Department of Obstetrics and Gynecology, Attikon University Hospital, Athens, GRC; 2 2nd Department of Obstetrics and Gynecology, Aretaieion University Hospital, Athens, GRC; 3 2nd Department of Obstetrics and Gynaecology, Aretaieion University Hospital, Athens, GRC

**Keywords:** asherman’s syndrome, chronic endometritis, endometrial receptivity, in vitro fertilization, platelet-rich plasma, repeated implantation failure, thin endometrium

## Abstract

Implantation failure remains a major challenge in assisted reproductive technology, particularly in women with thin endometrium, repeated implantation failure (RIF), Asherman’s syndrome (AS), or chronic endometritis (CE). In recent years, autologous intrauterine platelet-rich plasma (PRP) has been proposed as a regenerative approach aimed at improving endometrial receptivity through the local release of growth factors, cytokines, and angiogenic mediators.

A narrative review of the literature published between 2015 and 2021 was performed using PubMed, Google Scholar, and Web of Science. Studies assessing intrauterine PRP administration in women undergoing assisted reproduction were included. Due to the limited availability of high-quality randomized evidence, data from a wide range of study designs-including randomized controlled trials, observational studies, pilot studies, and case reports-were reviewed in order to reflect current clinical experience.

Reported outcomes included endometrial thickness, implantation rate, clinical pregnancy rate, and live birth rate in patients with thin endometrium, RIF, AS, and CE. Most studies described an association between PRP administration and improvements in endometrial thickness, vascularity, implantation, and clinical pregnancy rates. In women with thin endometrium, PRP was frequently associated with an increase in endometrial thickness to at least 7 mm and improved reproductive outcomes. In cases of repeated implantation failure, some randomized controlled trials suggested higher pregnancy and implantation rates in PRP-treated patients compared with controls. Evidence regarding AS and CE was largely derived from case reports and small case series, reporting enhanced endometrial regeneration and occasional successful pregnancies.

Overall, intrauterine PRP appears to be a promising and well-tolerated autologous intervention for women with poor prognostic factors. Nevertheless, the available evidence remains preliminary, and further large-scale randomized controlled trials using standardized PRP preparation and administration protocols are required to clarify its effectiveness and define its role in evidence-based assisted reproduction.

## Introduction and background

Implantation failure remains one of the major barriers to successful fertility treatment, even in cycles where embryo quality is considered optimal. Although advances in in vitro fertilization (IVF) have markedly improved laboratory outcomes, a substantial proportion of patients continue to experience suboptimal clinical results. Increasing evidence suggests that these failures are more often related to impaired endometrial receptivity rather than intrinsic embryonic incompetence [1-3].

Thin endometrium, repeated implantation failure (RIF), intrauterine adhesions such as Asherman’s syndrome (AS), and chronic endometritis (CE) represent some of the most common uterine conditions associated with reduced implantation potential and lower success rates in assisted reproductive technology (ART). Collectively, these conditions highlight the endometrium as an active and dynamic tissue whose functional capacity depends on the tightly regulated interaction of hormonal signaling, vascular development, inflammatory balance, and immune modulation.

Endometrial receptivity develops within a narrow “window of implantation,” during which synchronized molecular changes allow efficient embryo apposition, adhesion, and invasion. Disruptions in angiogenesis, cytokine signaling, stromal decidualization, or epithelial integrity may compromise this window and diminish implantation potential despite adequate hormonal priming [4]. Conventional therapeutic strategies-such as estrogen supplementation, vasodilators, granulocyte colony-stimulating factor (G-CSF), intrauterine perfusions, or hysteroscopic adhesiolysis-often yield inconsistent or limited results in women with refractory endometrial dysfunction.

Platelet-rich plasma (PRP) has emerged as a promising biological therapy due to its high concentrations of autologous growth factors and cytokines, including vascular endothelial growth factor (VEGF), platelet-derived growth factor (PDGF), epidermal growth factor (EGF), transforming growth factor-β (TGF-β), and insulin-like growth factor (IGF) [5,6]. These bioactive molecules are known mediators of angiogenesis, extracellular matrix remodeling, cell proliferation, and inflammatory modulation-mechanisms that are directly relevant to endometrial regeneration and receptivity.

Early clinical evidence suggests that intrauterine PRP infusion may enhance endometrial proliferation, promote neovascularization, and improve endometrial quality, thereby increasing the probability of implantation. Several observational studies and small interventional trials in women with thin endometrium or RIF have reported improvements in endometrial thickness, vascularity, and clinical pregnancy rates following PRP administration [7-10]. Preliminary data also indicate potential benefits in patients with AS and CE, and where PRP may contribute to tissue regeneration and restoration of endometrial function even in cases refractory to standard therapies [11-13].

Despite encouraging results, the current body of evidence is limited by variability in PRP preparation protocols, platelet concentration, leukocyte content, infusion timing, and outcome definitions. Such heterogeneity complicates comparison across studies and underscores the need for standardized methodologies and larger randomized controlled trials. This narrative review summarizes the available clinical evidence on intrauterine PRP use within ART, discusses its proposed biological mechanisms, and identifies key areas requiring further research to clarify clinical efficacy and optimize implementation.

## Review

PRP has emerged as a promising autologous biological therapy in reproductive medicine due to its high concentration of platelets enriched with cytokines, chemokines, and growth factors with regenerative, angiogenic, and immunomodulatory properties [2-4]. PRP contains bioactive molecules such as VEGF, PDGF, TGF-β, IGF, and EGF-factors known to promote angiogenesis, stromal proliferation, extracellular matrix remodeling, and a shift toward an implantation-favorable microenvironment within the endometrium [1,4]. Experimental evidence further suggests that PRP may activate dormant follicles through modulation of ovarian signaling pathways, mitochondrial activity, and local growth factor expression, positioning PRP as a potential adjunct in diminished ovarian reserve and ovarian rejuvenation research [5]. A key advantage of PRP is its autologous origin, which minimizes immunological risk and allows its use across diverse infertility etiologies. By delivering concentrated growth factors directly to the target tissue, PRP supports angiogenesis, enhances cellular proliferation and migration, reduces inflammation, and facilitates tissue repair-mechanisms particularly relevant to patients with thin endometrium, RIF, CE, and intrauterine adhesions.

PRP preparation across clinical studies follows broadly comparable principles. Typically, 10-18 mL of autologous venous blood undergoes single- or double-spin centrifugation to separate red blood cells, platelet-poor plasma, and the platelet-enriched fraction [4]. The resulting 0.5-1 mL of PRP generally contains platelet concentrations two- to three-fold higher than baseline values, although considerable variability exists among studies. Activation protocols differ (calcium chloride, thrombin, or no activator), reflecting a lack of standardized processing, one of the main contributors to heterogeneity in reported outcomes [3,4]. Despite methodological variations, the majority of PRP trials report improvements in endometrial thickness, endometrial vascularity, implantation rate, and clinical pregnancy rate across several indications [6-17].

Before presenting the available evidence across different reproductive indications, the key study characteristics, PRP protocols, and outcomes described in the literature are summarized in Table 1, which provides an overview of major clinical studies evaluating intrauterine PRP in ART [6-17].

**Table 1:** Summary of major clinical studies evaluating intrauterine platelet-rich plasma (PRP) in assisted reproductive technology (ART). RIF: repeated implantation failure; RCT: randomized controlled trial; G-CSF: granulocyte colony-stimulating factor; PRP: platelet-rich plasma; AS: Asherman’s syndrome.

Year	Study	Patients	Indication	PRP Protocol	Clinical Pregnancy Rate	Outcome Summary
2015	Chang et al. [6]	Small Pilot Cohort	Thin Endometrium	0.5–1 mL on Day 10	100%	Normalization of endometrial thickness with a high pregnancy rate
2018	Eftekhar et al. [7]	83	Thin Endometrium	0.5 mL on Day 13	32.5%	All reached >7 mm thickness
2020	Agarwal et al. [10]	32	Thin Endometrium	Hysteroscopic instillation	32.5%	All achieved >7 mm
2020	Zamaniyan et al. [11]	98	RIF	Before transfer	48.3%	RCT; significant improvement
2019	Mehrafza et al. [12]	123	RIF	Before transfer	Higher than G-CSF	PRP outperformed the comparator
2021	Nazari et al. [13]	60	RIF	Before transfer	53.06% vs 27.08%	Reduced inflammation and improved pregnancy rate
2021	Puente et al. [16]	1	AS	Days 12 & 15	Case Report	Successful pregnancy
2018	Aghajanova et al. [17]	2	AS	After surgery	Case Report	Endometrial recovery and live birth

Patients with persistently thin endometrium represent one of the most challenging groups in assisted reproduction, as inadequate endometrial thickness is strongly associated with reduced implantation rates, increased cycle cancellation, and poor clinical outcomes. Multiple studies have evaluated the role of PRP as an adjunctive therapy in this population, and the accumulating evidence suggests a consistent beneficial effect. Chang et al. reported remarkable results in one of the earliest studies, noting a 100% clinical pregnancy rate following intrauterine infusion of 0.5-1 mL of PRP in women with refractory thin endometrium, along with normalization of endometrial thickness in all participants [6]. Eftekhar et al., in a larger prospective study, found that PRP significantly improved endometrial thickness and yielded higher clinical pregnancy rates compared with standard hormonal therapy alone [7]. Agarwal et al. demonstrated similar improvements, reporting that all women in their cohort achieved an endometrial thickness of >7 mm after PRP administration [10].

Additional evidence from Wang et al. supports the mechanistic basis of these findings, showing that PRP enhances endometrial stem cell proliferation, improves angiogenic signaling, and promotes molecular changes associated with receptivity [8]. Most protocols administered 0.5-1 mL of PRP between cycle days eight and 17, with considerable variation in timing and frequency across studies. Collectively, the available evidence supports PRP as a promising intervention for women with refractory thin endometrium, especially when conventional therapies fail to induce adequate endometrial growth.

RIF represents a complex clinical condition in assisted reproductive technology, characterized by persistent failure to achieve pregnancy despite the transfer of morphologically high-quality embryos. Increasing evidence suggests that impaired endometrial receptivity, rather than embryonic factors alone, plays a central role in its pathophysiology, involving altered decidualization, dysregulated immune responses, and insufficient angiogenesis at the implantation site. In this context, PRP has attracted interest as a potential adjunctive therapy due to its regenerative and immunomodulatory properties. Several randomized controlled trials and prospective studies have explored the role of intrauterine PRP administration in women with RIF. Zamaniyan et al. reported higher chemical and clinical pregnancy rates following PRP infusion before embryo transfer compared with standard management [11]. In a comparative study, Mehrafza et al. observed improved implantation and pregnancy outcomes with PRP compared with granulocyte colony-stimulating factor (G-CSF) [12]. Similarly, Nazari et al. described increased clinical pregnancy rates after PRP treatment, suggesting a potential modulatory effect on the endometrial microenvironment [13].

The proposed mechanisms underlying the potential efficacy of PRP in recurrent implantation failure include enhanced decidualization, increased stromal cell proliferation, promotion of local angiogenesis, and modulation of immune responses at the maternal-fetal interface [11-14]. Importantly, several of these pathways overlap with the pathophysiological mechanisms implicated in CE, a condition frequently associated with RIF. Persistent low-grade endometrial inflammation, altered cytokine expression, impaired vascular remodeling, and dysregulated uterine natural killer (uNK) cell activity have all been described in both CE and RIF, contributing to a non-receptive endometrial microenvironment. Growth factors contained in PRP, including PDGF, VEGF, and transforming growth factor-β (TGF-β), may help restore these altered molecular pathways by enhancing vascularization, supporting endometrial regeneration, and attenuating local inflammatory responses. Through its immunomodulatory effects, PRP may also favorably influence uNK cell activity and promote the establishment of a more tolerant implantation environment. Collectively, these mechanisms provide a biological rationale for the use of PRP as a potential adjunctive therapy in patients with RIF, particularly in those with coexisting inflammatory endometrial conditions.

AS is characterized by intrauterine adhesions and varying degrees of endometrial fibrosis resulting from damage to the basal layer of the endometrium, most commonly following uterine curettage or intrauterine infection [10]. These pathological changes impair endometrial regeneration, vascularization, and receptivity, and are associated with menstrual disturbances, infertility, and recurrent implantation failure [11]. Hysteroscopic adhesiolysis remains the cornerstone of treatment; however, a substantial proportion of patients continue to exhibit a persistently thin or hyporeceptive endometrium following surgery, and effective adjunctive therapeutic options in this setting remain limited.

Experimental data suggest that PRP may enhance endometrial recovery by increasing stromal cell proliferation, promoting angiogenesis, activating genes involved in endometrial regeneration, and reducing fibrosis through the modulation of matrix metalloproteinases and anti-fibrotic pathways [16,17]. Clinical evidence, which is largely derived from case reports and small case series, is nevertheless encouraging. Puente-Gonzalo et al. described a case of severe refractory AS in which intrauterine PRP administration on cycle days 12 and 15 was associated with improved endometrial development and a subsequent successful pregnancy [16]. Similarly, Aghajanova et al. reported two cases in which PRP, administered following hysteroscopic adhesiolysis, resulted in measurable improvements in endometrial thickness, vascularity, and histological appearance, ultimately leading to clinical pregnancies and live births despite persistently thin endometrium [17].

Taken together, the available evidence, although preliminary, suggests that PRP may represent a useful adjunctive strategy in selected patients with AS, particularly in cases with persistent endometrial dysfunction following standard surgical management. Future prospective studies are needed to better define patient selection criteria, treatment protocols, and long-term reproductive outcomes.

CE is a persistent inflammatory condition of the endometrium that disrupts implantation, impairs decidualization, and alters immune homeostasis. CE is strongly associated with implantation failure, unexplained infertility, and adverse ART outcomes [18,19]. Although standard treatment relies on targeted antibiotic therapy, a considerable proportion of patients remain refractory, particularly those with concomitant RIF or underlying endometrial dysfunction. PRP has emerged as a promising adjunctive therapy due to its combined anti-inflammatory, regenerative, and immunomodulatory properties.

PRP contains bioactive molecules capable of modulating aberrant immune responses observed in CE. Growth factors and cytokines within PRP may downregulate chronic inflammatory pathways, enhance stromal cell remodeling, and restore normal decidualization. Mechanistically, PRP is thought to influence uterine natural killer (uNK) cell profiles, reduce endometrial fibrosis, and correct dysregulated cytokine expression, factors that contribute to CE-associated implantation failure.

Clinical evidence supporting the use of PRP in inflammatory endometrial conditions, including CE, remains limited but encouraging. Studies conducted primarily in women with repeated implantation failure have reported improved clinical pregnancy outcomes following intrauterine PRP administration, suggesting potential immunomodulatory effects that may also be relevant in inflammatory endometrial disorders [13]. In this context, Sfakianoudis et al. described successful implantation and live birth in a patient with refractory CE and repeated implantation failure following PRP treatment, indicating a possible role in restoring endometrial receptivity when antibiotic therapy alone is insufficient [14]. Additional case reports and small series, including the study by Li et al., have reported improvements in endometrial thickness, resolution of inflammatory features, and successful pregnancies after PRP administration in patients with persistent CE [15].

Despite these promising observations, interpretation of the current literature is limited by substantial heterogeneity across studies, particularly with respect to PRP preparation protocols, platelet concentration, leukocyte content, dosing frequency, timing of administration relative to the menstrual cycle or embryo transfer, and route of delivery. This methodological variability complicates direct comparison of outcomes and precludes firm evidence-based recommendations. Accordingly, larger well-designed randomized controlled trials using standardized PRP protocols are required to clarify efficacy, optimize treatment parameters, and define the clinical role of PRP alongside established therapeutic strategies.

**Figure 1:**
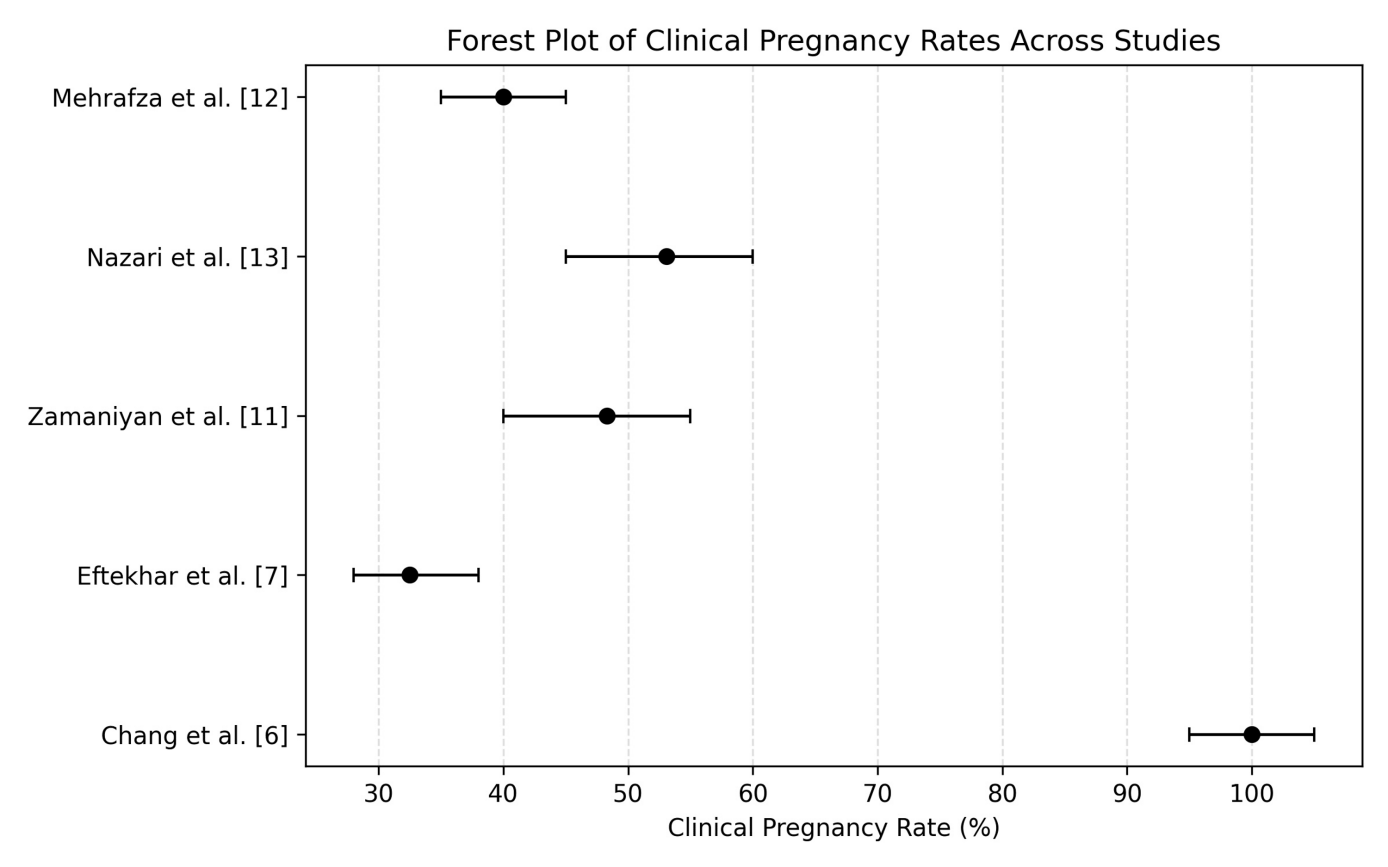
Forest plot summarizing clinical pregnancy rates reported across studies evaluating intrauterine platelet-rich plasma (PRP) in assisted reproductive technology (ART). The plot includes data from [6, 7, 11-13]

The aggregated clinical pregnancy outcomes reported across PRP studies are summarized in Figure 1, highlighting both overall trends and the variability observed among different patient populations and study designs. Collectively, these findings support the biological plausibility of PRP as a therapeutic adjunct in CE by addressing inflammatory dysregulation and promoting endometrial repair.

The use of PRP in reproductive medicine has gained increasing attention due to its regenerative, angiogenic, and immunomodulatory properties, which directly target several mechanisms implicated in endometrial dysfunction and implantation failure. Across the available literature, PRP appears to enhance endometrial thickness, vascularization, and receptivity markers, contributing to improved implantation and clinical pregnancy rates in individuals who have traditionally faced limited treatment options. Although the overall evidence remains preliminary, the consistency of reported benefits across multiple infertility etiologies, thin endometrium, RIF, CE, and AS, supports the biological plausibility of PRP as a meaningful adjunct in ART.

The therapeutic effects of PRP are mediated through a high concentration of autologous growth factors and cytokines, including VEGF, PDGF, EGF, IGF, and TGF-β, which play central roles in angiogenesis, extracellular matrix remodeling, stromal proliferation, and reduction of inflammation [4]. These pathways are essential for establishing a receptive endometrial environment. Experimental studies indicate that PRP dynamically alters gene expression, upregulates implantation-associated markers, enhances endometrial stem cell activity, and modulates immune pathways, including uterine natural killer (uNK) cell function, suggesting that its benefits extend beyond simple mechanical enhancement of endometrial thickness.

In patients with a thin endometrium, PRP has demonstrated the most consistent clinical effects. Multiple studies, including those by Chang et al. and Eftekhar et al., reported significant increases in endometrial thickness and restoration of implantation potential in individuals who previously failed to achieve adequate endometrial development despite maximal hormonal therapy [6,7]. These findings align with mechanistic research showing increased angiogenesis and stromal proliferation following PRP exposure. The effect of PRP in RIF also appears promising. Randomized controlled trials by Zamaniyan et al. and Mehrafza et al. demonstrated significantly higher pregnancy outcomes among PRP-treated patients compared with controls or alternative therapies such as G-CSF [11,12]. Improvements in decidualization, immune tolerance at the maternal-fetal interface, and stromal remodeling have been proposed as mechanistic explanations. These results suggest that PRP may help correct subtle endometrial defects that are not readily detectable through standard imaging or hormonal testing.

In CE and AS, conditions characterized by chronic inflammation and fibrosis, PRP’s regenerative and anti-inflammatory properties may help restore normal tissue architecture and function. Case reports and small series describe improved endometrial thickness, inflammation resolution, and successful pregnancies following PRP administration [15-17]. Although such evidence remains low in the hierarchy of clinical data, the consistency of biological responses and positive outcomes across multiple etiologies supports further investigation. A major limitation of the current literature is significant heterogeneity in PRP preparation techniques, platelet concentrations, leukocyte content, activation methods, dosing volumes, and timing of administration. These variations make cross-study comparison difficult and limit the ability to establish standardized clinical recommendations. Additionally, most available studies are small, single-center investigations, often uncontrolled or observational, with limited follow-up and variability in outcome definitions. Despite these challenges, the repeated demonstration of improved clinical outcomes suggests a genuine therapeutic signal that warrants more rigorous investigation.

The current literature on intrauterine PRP in ART is promising but remains limited by several important methodological and clinical constraints. A major limitation is the absence of standardized PRP preparation and administration protocols. Substantial heterogeneity exists across studies with respect to centrifugation methods, platelet concentration, leukocyte content, activation techniques, and final infusion volume, as well as dosing frequency and timing of administration. At present, no uniform infusion volume or dosing strategy has been established. These inconsistencies make it difficult to define the optimal formulation and hinder direct comparison of outcomes across studies. Additionally, most available evidence is derived from small, single-center cohorts, non-randomized trials, or case reports, particularly in CE and AS. The lack of adequately powered randomized controlled trials limits the generalizability of findings and the ability to draw firm conclusions regarding efficacy.

Another limitation is the variability in outcome measures and follow-up duration. Many studies focus primarily on endometrial thickness and early clinical pregnancy, with limited reporting of ongoing pregnancy, live birth rates, obstetric outcomes, or long-term reproductive safety. Furthermore, PRP’s mechanism of action remains incompletely characterized at the molecular level, particularly with respect to its effects on immune modulation, angiogenesis, and endometrial stem cell populations. There is also no consensus regarding the optimal timing of PRP administration within the natural or stimulated cycle, and treatment protocols vary widely among centers.

Given these gaps, several recommendations can be made. Standardization of PRP preparation, including specification of platelet concentration, leukocyte content, activation method, infusion volume, and dosing frequency, is essential to improve reproducibility and comparability across studies. Future research should prioritize large, multicenter randomized controlled trials to assess PRP efficacy in thin endometrium, RIF, CE, and AS, using rigorous and clinically meaningful outcome measures. These should extend beyond implantation and clinical pregnancy rates to include live birth rate, obstetric complications (such as preterm birth, hypertensive disorders of pregnancy, placental abnormalities, and gestational diabetes), and long-term maternal and neonatal safety.

Future directions also include investigation into optimal dosing strategies, repeated or sequential PRP infusions, and combination therapies (e.g., PRP with stem cell-based treatments, granulocyte colony-stimulating factor, or hormonal modulation). In parallel, integration of mechanistic analyses and objective biomarkers-such as molecular markers of angiogenesis, inflammation, immune modulation, and endometrial receptivity, as well as transcriptomic profiles and imaging-based vascularity indices-may further enhance understanding of treatment response and patient selection. Overall, while PRP represents a promising regenerative adjunct in assisted reproductive technology, further well-designed studies are required to establish standardized protocols, clarify biological mechanisms, and define long-term clinical efficacy and safety across diverse patient populations.

## Conclusions

PRP has emerged as a biologically plausible and increasingly investigated therapeutic adjunct in ART. Current evidence, although still preliminary, suggests that PRP may meaningfully enhance endometrial repair, modulate local inflammatory and immune pathways, and improve endometrial receptivity in challenging clinical contexts such as thin endometrium, RIF, CE, and AS. Across these indications, reported improvements in endometrial thickness, vascular perfusion, and clinical pregnancy rates support the rationale for PRP as a potentially valuable intervention in women with persistently suboptimal endometrial characteristics despite standard management.

Nevertheless, the existing literature is characterized by methodological heterogeneity, limited sample sizes, inconsistent reporting of preparation protocols, and a scarcity of well-designed randomized controlled trials. These limitations preclude definitive conclusions regarding the magnitude of PRP’s therapeutic effect or its comparative effectiveness within ART. Future research must prioritize protocol standardization, robust trial design, and mechanistic studies capable of elucidating PRP’s molecular targets and identifying biomarkers predictive of treatment responsiveness. Clarifying these elements will be essential for integrating PRP into evidence-based reproductive practice and determining its true clinical utility in the management of complex endometrial dysfunction.
